# Retrospective Study of Traumatic Intra-Interspecific Interactions in Stranded Cetaceans, Canary Islands

**DOI:** 10.3389/fvets.2020.00107

**Published:** 2020-02-28

**Authors:** Raquel Puig-Lozano, Antonio Fernández, Pedro Saavedra, Marisa Tejedor, Eva Sierra, Jesús De la Fuente, Aina Xuriach, Josué Díaz-Delgado, Miguel Antonio Rivero, Marisa Andrada, Yara Bernaldo de Quirós, Manuel Arbelo

**Affiliations:** ^1^Veterinary Histology and Pathology, Atlantic Center for Cetacean Research, University Institute of Animal Health and Food Safety (IUSA), Veterinary School, University of Las Palmas de Gran Canaria, Canary Islands, Spain; ^2^Department of Mathematics, University of Las Palmas de Gran Canaria, Las Palmas of Gran Canaria, Spain; ^3^TVMDL, Texas A&M, Veterinary Medical Diagnostic Laboratory, Amarillo, TX, United States

**Keywords:** tooth-rake marks, social interaction, prey interaction, dolphin, agression, trauma

## Abstract

Aggressive encounters involving cetacean species are widely described in the literature. However, detailed pathological studies regarding lesions produced by these encounters are scarce. From January 2000 to December 2017, 540 cetaceans stranded and were necropsied in the Canary Islands, Spain. Of them, 24 cases of eight species presented social traumatic lesions produced by cetaceans of the same or different species. All the cases presented severe multifocal vascular changes, 50% (12/24) presented fractures affecting mainly the thoracic region, 41.7% (10/24) acute tooth-rake marks, 37.5% (9/24) undigested food in the stomach, 33.3% (8/24) tracheal edema, and 12.5% (3/24) pulmonary perforation. In 10 cases with tooth-rake marks, the distance between the teeth, allowed us to further identify the aggressor species: four cases were compatible with killer whales (*Orcinus orca*) affecting three species [pigmy sperm whale (*Kogia breviceps*), Cuvier's beaked whale (*Ziphius cavirostris*), and short-finned pilot whale (*Globicephala macrorhynchus*)] and four cases compatible with common bottlenose dolphins (*Tursiops truncatus*) affecting two species [short-beaked common dolphin (*Delphinus delphis*) and Atlantic spotted dolphin (*Stenella frontalis*)]. We also described two cases of intraspecific interaction in stripped dolphin (*Stenella coeruleoalba*). Microscopically, 70.8% (17/24) of the cases presented acute degenerative myonecrosis, 66.7% (14/21) presented vacuoles in the myocardiocytes, 36.8% (7/19) pigmentary tubulonephrosis, 31.6% (6/19) cytoplasmic eosinophilic globules within hepatocytes, 21.4% (3/14) hemorrhages in the adrenal gland, and 17.3% (4/23) bronchiolar sphincter contraction. The statistical analysis revealed that deep divers, in good body condition and nearby La Gomera and Tenerife were more prone to these fatal interactions. Additionally, in this period, three animals died due to an accident during predation: a false killer whale (*Pseudorca crassidens*) died because of a fatal attempt of predation on a stingray, and two Risso's dolphins (*Grampus griseus*) died as a consequence of struggling while predating on large squids.

## Introduction

The term “intra-interspecific interactions” refers to interaction with individuals of the same species (intraspecific) or with other species (interspecific). When the interactions become aggressive it may lead to serious injuries and/or death of the animal (1–4).

Social intra-interspecific interactions can produce mild multifocal lesions over the skin known as “tooth-rake marks” (external linear and parallel erosions on the skin inflicted by teeth), frequently observed healed in stranded animals. However, when interactions became aggressive, tooth-rake marks could be severe and ulcerate the skin affecting the subcutaneous and muscle tissue. Other lesions that had been reported in fatal encounters include: blunt traumas with subcutaneous focal or multifocal extensive hemorrhages, hematomas, tearing of the blubber, vertebral and/or ribs fractures, myonecrosis, tearing of the parietal pleura with associated-pulmonary hemorrhages, hemothorax, retroperitoneal hemorrhages, perforation of the abdominal wall, and liver rupture (3–6). Histologically, acute monophasic degeneration and hemorrhages in the muscle are common findings (4, 7). Pulmonary fat emboli (8) and myo-/hemoglobinuric nephrosis (4) can be observed by specific staining.

Aggressive encounters involving individuals of the same species are largely described in the literature [e.g., (9, 10)], including the formation of male alliances (11–13). Male alliances are responsible for violent kidnappings (“herding events”) of non-pregnant females to increase their mating opportunities as well as of infanticides in different cetacean species such as the Amazon river dolphin (*Inia geoffrensis*) (14), Indo-Pacific humpback dolphin (*Sousa chinensis*) (15), killer whale (16), tucuxi dolphin (*Sotalia guianensis*) (17), and bottlenose dolphin (*Tursiops* spp.) (1, 2, 18, 19). In Mysticetes, males humpback whales (*Megaptera novaeangliae*) have been reported escorting receptive females and threatening other males by thrashing of their flukes or signing as communication signals in the context of male competition (20). Although male coalitions have also been observed in whales, aggressive reactions are not usual, and fights rarely result in serious injury or death (10).

Violent interspecific interactions with other species may occur for reasons other than sexual competition, such as prey competition (21), fight practice (6), or predation on cetaceans and non-cetacean species. Killer whales (*Orcinus orca*) have been observed attacking or harassing about 20 different species of cetaceans, including both, odontocetes and Mysticetes (22–28). In addition, false killer whales (*Pseudorca crassidens*) predate on species of the genus *Stenella* spp. and short-beaked common dolphins (29).

The Canarian waters are known for their particular oceanographic features and their enormous diversity of cetacean species, with 30 species described so far (Banco de Datos de Biodiversidad de Canarias), some of them regularly seen year-round (30). Although there is evidence of habitat partitioning in the waters used by several cetacean species in La Gomera (31), most species coexist in other areas of the Canary Islands. This confluence is motivated by factors such as temperature, deep waters near the coast, an abundance of food resources, and calm waters in southwestern regions. Thus, numerous interactions between different cetacean species inhabiting these waters are expected.

This study aims to investigate the prevalence and the pathologic findings associated with social traumatic interactions between cetacean species and foraging fatalities in the Canary Islands, based on postmortem examinations.

## Materials and Methods

Post-mortem examinations following standardized protocols (32) were carried out on 540 stranded cetaceans in Canary Islands, Spain, from 2000 to 2017. Required permission for the management of stranded cetaceans was issued by the environmental department of the Canary Islands' Government and the Spanish Ministry of Environment. Experiments on live animals were not performed.

Epidemiology of the stranding (i.e., location and date), life history data (i.e., species, age class, sex, gonad maturation), and body condition were systematically recorded following standardized protocols (33). Age class (i.e., neonate, calf, juvenile, subadult, and adult) was established based on total body length (20), histologic gonadal examinations (33), and in some cases, osteological studies (34). Body condition was estimated based on the external physical conformation (the degree of epiaxial concavity or convexity, nuchal depression, the visibility of the ribs and vertebral transverse processes, as well as the presence or absence of nuchal and epicardial fat) in very poor, poor, fair and good body condition (35). For decomposition status, five codes were applied following IJsseldijk (36) classification: very fresh (code 1), fresh (code 2), moderate autolysis (code 3), advanced autolysis (code 4), or very advanced autolysis (code 5).

External and internal lesions were fully described, photographed, and sampled. Tissue samples were immersed in 10% neutral buffered formalin, routinely processed, embedded in paraffin, processed, sectioned at 5 μm and stained with hematoxylin and eosin for histopathologic analysis.

For the diagnosis of traumatic intra-interspecific interactions we took a conservative approach based on previous references (5, 6, 37) excluding the cases in which other possible traumatic causes of death such as fisheries interaction, vessel collision, or a live stranding (38–42) could not be ruled out. Stress-related lesions were histologically studied in selected samples upon availability [skeletal muscle (*n* = 24), lung (*n* = 23), cardiac muscle (*n* = 21), liver (*n* = 19), kidney (*n* = 19), and adrenal gland (*n* = 14)].

To identify factors associated with death due to intra-interspecific interaction between cetaceans (*n* = 24), categorical variables (i.e., species, diving behavior, age, sex, maturity, location, and body condition) were expressed as frequencies and percentages and were compared, as appropriate, using the Chi-square (χ^2^) test or the exact Fisher test. For statistical purposes, age classes were regrouped in neonate/calves, juvenile/subadults, and adults; and body condition categories were regrouped in poor/very poor and good/fair. Stranding locations were also regrouped based on geographical proximities and the presence of high-site fidelity populations: Eastern islands (Fuerteventura and Lanzarote), Western Islands (El Hierro and La Palma), La Gomera and Tenerife, and Gran Canaria. Statistical significance was set at *p* < 0.05. Data were analyzed using the R package, version 3.3.1 (43).

## Results

Between January 2000 and December 2017, a total of 540 cetaceans stranded along the coasts of the Canarian archipelago were necropsied. A pathological entity (category of cause of death) was identified in 432 cases. Of them, 27 individuals (6.3%) presented severe lesions consistent with aggressive intra-interspecific interactions. In 88.9% (24/27) of the cases, social traumatic interactions between cetaceans of the same or different species produced blunt-force traumas that led to death. In 11.1% (3/27) of the cases, the animals died due to fatal accidents while foraging on potential prey (squid or stingray) (Table 1). Two out of 27 affected cetaceans were found stranded alive (case no 14 and 18).

**Table 1:** Twenty-seven cetaceans dead due to traumatic intra-interspecific interaction the Canary Islands (from January 2000 to December 2017), between cetaceans (*n* = 24) or because a failure in the predation (*n* = 3).

**Case**	**Species**	**Diving behavior**	**Stranding date**	**Island**	**Stranding event**	**Sex**	**Age**	**Body condition**	**Decomposition state**	**Sexual maturity**	**Traumatic behavior**
1	*Globicephala macrorhynchus*	D	14.07.2003	FV	D	F	Adult	2	2	M	S
2	*Mesoplodon europaeus*	D	08.09.2003	TNF	D	M	Calf	2	3	I	S
3	*Stenella frontalis*	S	09.06.2004	TNF	D	M	Neonate	2	3	I	S
4	*Stenella coeruleoalba*	S	05.02.2005	LNZ	D	F	Adult	2	2	M	S
5	*Stenella coeruleoalba*	S	14.06.2005	TNF	D	F	Juvenile	2	3	I	S
6	*Kogia breviceps*	D	31.03.2006	LG	D	F	Adult	2	3	M	S
7	*Mesoplodon europaeus*	D	28.07.2006	TNF	D	M	Calf	2	3	I	S
8	*Globicephala macrorhynchus*	D	30.11.2006	GC	D	M	Adult	ND	4	M	S
9	*Kogia breviceps*	D	06.04.2007	TNF	D	F	Adult	2	3	M	S
10	*Kogia breviceps*	D	29.08.2007	LNZ	D	F	Juvenile	1	3	M	S
11	*Globicephala macrorhynchus*	D	07.09.2007	TNF	D	M	Neonate	ND	5	I	S
12	*Delphinus delphis*	S	14.01.2008	TNF	D	M	Calf	2	3	I	S
13	*Delphinus delphis*	S	08.03.2008	TNF	D	M	Calf	2	3	I	S
14	*Pseudorca crassidens*	S	11.03.2008	LNZ	A	M	Calf	1	2	I	P
15	*Delphinus delphis*	S	09.07.2008	FV	D	M	Calf	1	4	I	S
16	*Stenella coeruleoalba*	S	09.02.2009	GC	D	F	Calf	2	2	I	S
17	*Grampus griseus*	D	06.03.2009	FV	D	M	Subadult	2	2	I	P
18	*Globicephala macrorhynchus*	D	06.07.2009	TNF	A	M	Subadult	2	2	M	S
19	*Stenella frontalis*	S	13.04.2010	TNF	D	F	Adult	2	2	M	S
20	*Grampus griseus*	D	17.09.2010	TNF	D	F	Adult	1	2	M	P
21	*Tursiops truncatus*	S	05.08.2011	TNF	D	F	Calf	ND	4	I	S
22	*Globicephala macrorhynchus*	D	24.08.2011	FV	D	F	Calf	1	3	I	S
23	*Stenella frontalis*	S	19.03.2013	TNF	D	F	Adult	2	3	M	S
24	*Globicephala macrorhynchus*	D	16.06.2013	TNF	D	M	Calf	2	4	I	S
25	*Globicephala macrorhynchus*	D	25.02.2015	LNZ	D	F	Juvenile	2	4	M	S
26	*Globicephala macrorhynchus*	D	20.05.2015	TNF	D	M	Adult	2	4	M	S
27	*Ziphius cavirostris*	D	22.05.2017	GC	D	M	Adult	ND	4	ND	S

*The table shows the species, diving behavior (D, deep diver; S, shallow diver), stranding date (day.month.year), location (FV, Fuerteventura; GC, Gran Canaria; LG, La Gomera; LNZ, Lanzarote; TNF, Tenerife), the type of stranding event (D, death; A, alive), sex (F, female; M, male); age (neonate, calf, juvenile, subadult, adult) of each case (n = 27). Forensic studies allow us to know the following data: body condition (1: poor/very poor; 2: good/fair), decomposition state (2: fresh; 3: moderate autolysis; 4: advanced autolysis), sexual maturity (I, immature; M, mature), and the traumatic behavior that cause the death of the animal (S: social traumatic interaction between cetaceans of the same species or other; P: death due to an accident during predation)*.

## Social Traumatic Interactions Between Cetaceans

### Gross Findings

**Figure 1:**
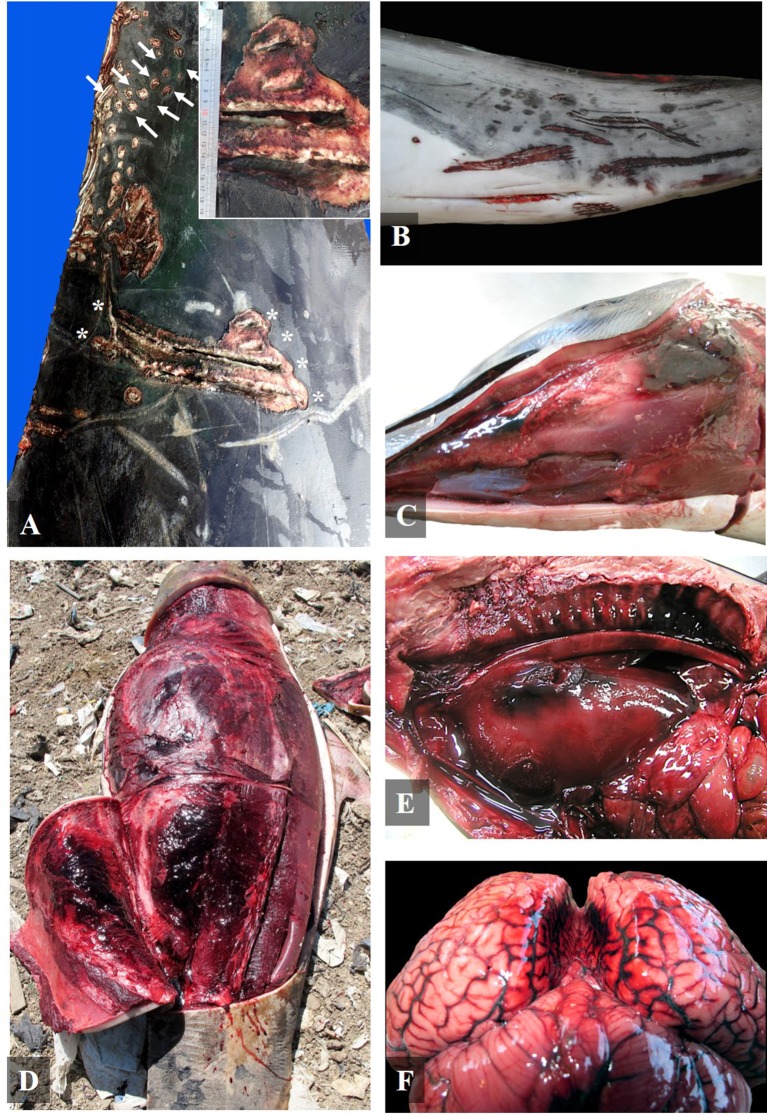
Gross findings of social traumatic intra-interspecific interaction between cetaceans. **(A)** Right lateral view of the peduncle of an adult Cuvier's beaked whale with severe multifocal tooth-rake marks compatible with killer whale behind the dorsal fin (asterisk and inset) and along the dorsal part of the peduncle (arrows) (case 27); **(B)** Left ventrolateral view of the peduncle of a mature female striped dolphin with severe multifocal intraspecific tooth-rake marks (case 4); **(C)** Left ventrolateral view of head of a striped dolphin calf with a severe multifocal hematoma in the submandibular region (case 16); **(D)** Left ventrolateral view of a calf short-finned pilot whale with a subcutaneous and muscular hematoma in the abdominal region (case 24); **(E)** Left ventrolateral view of the thoracic cavity of a neonate of Atlantic spotted dolphin with a severe hemothorax associated to a perforation of the pleural and pulmonary parenchyma of the left lung, related to focally extensive hemorrhage on the adventitia of the aorta and in the rete mirabile (case 3); and **(F)** Caudal view of the brain of an Atlantic spotted dolphin adult with a severe diffuse vascular congestion of the meninges and bilateral hemorrhages between brain hemispheres (case 23).

All the animals diagnosed with intra-interspecific trauma (24/24) presented multifocal severe vascular changes such as hemorrhages in the blubber; 62.5% (15/24) presented hemorrhages and/or congestion in the central nervous system (Figure 1F); 54.2% (13/24) presented subcutaneous hematomas (Figures 1C,D); 50% (12/24) presented hemothorax; 29.2% (7/24) presented hemoabdomen, and 4.2% (1/24) presented hemopericardium (Table 2).

**Table 2:** Macroscopic findings in cases of social traumatic intra-interspecific interaction between cetaceans (*n* = 24).

**Case**	**Species**	**Interspecific tooth-rake marks**	**Intraspecific tooth-rake marks**	**Healed rake marks**	**Skin erosion/laceration**	**Skin vascular changes**	**Postmortem shark bites**	**Hematomas**	**Fractures**					**Hemothorax**	**Hemoabdomen**	**Hemoapericardium**	**Lung perforation**	**Non-digested food**	**Tracheal edema**	**CNS vascular changes**
									**Cranium**	**Mandibles**	**Spine**	**Ribs**	**Scapula**							
1	*Globicephala macrorhynchus*	N	N	Y	N	Y	N	Y	N	N	N	N	N	N	N	N	N	N	Y	N
2	*Mesoplodon europaeus*	N	N	Y	N	Y	N	N	Maxilla	S (L)	M (T)	M (R & L)	S (L)	Y	Y	N	N	Y	N	Y
3	*Stenella frontalis*	N	N	N	N	Y	N	N	N	N	N	N	N	Y	N	N	Y	N	N	Y
4	*Stenella coeruleoalba*	N	Y	Y	N	Y	N	Y	N	N	N	M (R)	N	N	N	N	N	Y	Y	Y
5	*Stenella coeruleoalba*	N	Y	Y	N	Y	N	Y	N	N	N	M (L: 5°,6°,7°)	N	N	N	N	N	N	N	Y
6	*Kogia breviceps*	N	N	Y	Y	Y	N	Y	N	N	N	N	N	N	N	N	N	Y	Y	Y
7	*Mesoplodon europaeus*	N	N	Y	Y	Y	N	N	N	N	N	N	N	Y	Y	N	N	Y	N	N
8	*Globicephala macrorhynchus*	N	N	Y	N	Y	Y	N	N	N	N	N	N	Y	N	N	N	Y	N	N
9	*Kogia breviceps*	Y	N	Y	N	Y	Y	Y	N	N	N	N	N	N	Y	N	N	Y	Y	Y
10	*Kogia breviceps*	Y	N	Y	N	Y	Y	Y	N	N	N	N	N	N	N	N	N	N	Y	Y
11	*Globicephala macrorhynchus*	N	N	Y	N	Y	N	Y	N	N	N	M (R & L)	N	Y	N	N	Y	N	N	N
12	*Delphinus delphis*	Y	N	Y	N	Y	N	N	N	N	N	M (L: 5)	N	Y	N	N	N	N	N	N
13	*Delphinus delphis*	Y	N	Y	N	Y	N	N	N	N	N	M (L: 6)	N	N	N	N	N	N	N	Y
15	*Delphinus delphis*	Y	N	Y	N	Y	N	N	N	N	N	M (L: 3)	N	N	N	N	N	N	N	N
16	*Stenella coeruleoalba*	N	N	Y	Y	Y	N	N	N	N	N	N	N	N	N	N	N	N	Y	Y
18	*Globicephala macrorhynchus*	N	N	Y	N	Y	N	N	Right tympanic and bones of temporal region	N	N	N	N	N	N	N	N	N	Y	Y
19	*Stenella frontalis*	Y	N	Y	Y	Y	N	N	N	N	T (2 last) L (3 proximal)	S (5°)	N	N	N	N	N	N	N	Y
21	*Tursiops truncatus*	N	N	Y	N	Y	N	N	N	N	N	N	N	Y	Y	Y	N	N	N	Y
22	*Globicephala macrorhynchus*	Y	N	Y	Y	Y	Y	Y	N	N	N	N	N	N	Y	N	N	N	Y	Y
23	*Stenella frontalis*	N	N	Y	N	Y	N	N	N	N	T (5 last)	M (L: 3°, 4°, 5°, 6°, 7°, 8°, 9°, 10°, 11°, 12°)	N	Y	N	N	Y	Y	N	Y
24	*Globicephala macrorhynchus*	N	N	Y	N	Y	Y	Y	N	N	N	N	N	Y	Y	N	N	N	N	N
26	*Globicephala macrorhynchus*	N	N	Y	N	Y	Y	Y	N	N	N	M (L: 3°, 4°, 5°, 6°, 7°, 8°, 9°)	N	Y	N	N	N	Y	N	Y
27	*Ziphius cavirostris*	Y	N	Y	N	Y	N	N	N	N	N	N	N	Y	N	N	N	N	N	N
		8	2	23	6	24	7	11	2	2	3	11	1	12	7	1	3	9	8	15

*The cases in which the death was related to an accident during the predation are not included (cases 14, 17, and 20). The table shows the number of the case, the species, and the presence (Y, yes) or the absence (N, no) of intra-interspecific tooth-rake marks, healed rake marks, skin erosion/laceration, vascular changes in the skin, postmortem shark bites, subcutaneous and muscle hematomas, fractures [cranium, mandibles (S, single; M, multiple; R, right; and L, left), vertebrae (T, thoracic and L, lumbar), ribs (the exact number of rib fractures is given in case it was recorded, also the side), and scapula], hemothorax, hemoabdomen, hemopericardium, lung perforation, non-digested food, tracheal edema, and vascular changes and Central Nervous System (CNS)*.

Healed tooth-rake marks (linear non-severe parallel superficial skin lesions) compatible with social intraspecific behavior were observed in 95.8% of the cases (23/24). Severe acute multifocal tooth-rake marks were found in 41.7% of the cases (10/24) (Figures 1A,B). Tooth-rake marks were compatible with killer whale interaction in two pregnant female pigmy sperm whales (cases 9 and 10), a calf short-finned pilot whale (case 22), and an adult Cuvier's beaked whale (case 27). In these cases, 28–43 mm separation between tooth-rake marks was observed but also punctures (Figure 1A). The other four animals, three calves of short-beaked common dolphin (cases 12, 13, and 15) and one adult of Atlantic spotted dolphin (case 19), presented 7–12 mm separation tooth-rake marks compatible with adult bottlenose dolphin interaction (Table 3). Also, intraspecific tooth-rake marks were present in two female striped dolphins (cases 4 and 5), mainly found in genital area (Figure 1B) and head (Table 3).

**Table 3:** Distance between teeth in four species of small Odontocetes.

**Species**	**Intertooth spacing (mm)**	**Maximum distance between teeth (mm)**
*Tursiops truncatus*	7–12	15
*Globicephala macrorhynchus*	20–33	40
*Stenella coeruleoalba*	4–6	6
*Stenella frontalis*	5–6	6

*Data referred to osteological studies of adult cetaceans stranded in the Canary Islands (34)*.

Semi-circular parallel multifocal tooth marks without inflammatory or vascular changes in the tissue, mainly in the dorsal or ventral part of the peduncle close to the perineal area, consistent with post-mortem shark bites, were found in 29.2% of the animals (7/24) (cases 8, 9, 10, 22, 24, 25, and 26) (Table 2).

Half of the cases diagnosed with intra-interspecific trauma (12/24) presented bone fractures, and in all of these cases, the fractures involved multiple bones and were bilateral in 5 of them. The thorax was the most affected body region with fractures involving the ribs (cases 2, 4, 5, 11, 12, 13, 15, 19, 23, 25, and 26), thoracic vertebrae (cases 2, 19, and 23), and the scapula (case 2). Other bones were also fractured such as the mandible (cases 2 and 24), the maxilla (case 2), the tympanic and the bones of the temporal region (case 18), and the lumbar vertebrae (case 19). In the case of the ribs, multiple contiguous unilateral rib fractures were most often detected. Only one individual had a single rib fracture (case 19) (Table 2).

Other macroscopic findings observed were: undigested food in the stomach in 37.5% (9/24) of the cases (cases 2, 4, 6, 7, 8, 9, 23, 25, and 26); tracheal edema in 33.3% (8/24) of the cases (cases 1, 4, 6, 9, 10, 16, 18, and 22); and pulmonary perforations in 12.5% (3/24) of the cases (cases 3, 11, and 23) (Figure 1E, Table 2).

Finally, regarding sexually mature animals, three polytraumatized adult female pygmy sperm whales were pregnant (cases 6, 9, and 10). The stranding records of this species in the Canary Islands showed that 85.7% (6/7) of the mature females were also pregnant.

### Histological Findings

**Figure 2:**
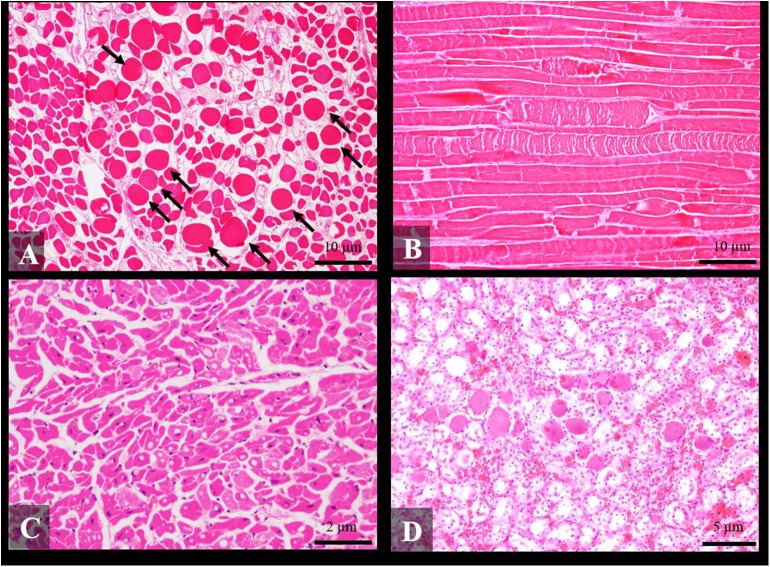
Histological findings of social traumatic intra-interspecific interaction between cetaceans stained routinely with hematoxylin-eosin. **(A)** Transversal section of *rectus abdominis* of an adult short-finned pilot whale with severe multifocal acute degenerative changes (hypercontraction) of muscle fibers (arrows) (case 26) × 10; **(B)** Longitudinal section of the *longissimus dorsi* of an adult Cuvier's beaked whale with severe multifocal segmental myodegeneration of muscle fibers (case 27) × 10; **(C)** Severe multifocal vacuolar degeneration in myocardiocites of a juvenile stripped dolphin (case 5) × 40; and **(D)** Pigmented intratubular casts in the kidney of an adult stripped dolphin (case 4) × 20.

Histological findings in skeletal muscle included mild to severe acute myonecrosis (segmental degeneration with hyalinized eosinophilic sarcoplasm and hypercontraction) in 70.8% (17/24) of the cases (Figures 2A,B). These lesions were severe in 29.2% (7/24) of the cases, moderate in 25% (6/24) and mild in 16.6% (4/24). Regarding cardiac muscle, degenerative changes such as juxtanuclear vacuolization and increased acidophilic cytoplasm of the myocardiocytes were present in 66.7% (14/21) of the cases (Figure 2C), being in 4.8% (1/21) of the cases severe, in 28.6% (6/21) moderate, and in 33.3% (7/21) mild.

With respect to the kidney, pigmentary tubulonephrosis with orange-red homogeneous intratubular casts was found in 36.8% (7/19) of the cases (Figure 2D). This finding was severe in 10.5% (2/19) of the cases, moderate in 5.3% (1/19), and mild in 21% (4/19).

In the case of the liver, intracytoplasmic hepatocellular hyaline globules were found in 31.6% (6/19) of the cases, being severe in 10.5% (2/19) of the cases, moderate in 5.3% (1/19), and mild in 16.8% (3/19) of the cases.

Other mild to moderate histological findings included corticomedullary adrenal hemorrhages in 21.4% (3/14) of the animals and bronchiolar sphincter contraction in 17.3% (4/23) of the cases.

### Statistical Analysis

#### Species

Eight different species presented lesions consistent with social traumatic intra-interspecific interaction (Table 4). The most affected species was the short-finned pilot whale with 33.3% of the cases (8/24); followed by the pygmy sperm whale, the short-beaked common dolphin, the striped dolphin and the Atlantic spotted dolphin with 12.5% of the cases each of them (3/24); Gervais' beaked whale with 8.3% of the cases (2/24); and Cuvier's beaked whale and common bottlenose dolphin with 4.2% of the cases each (1/24) (Table 4). The prevalence of traumatic intra-interspecific interaction was not statistically significant different between species (*p* = 0.111).

**Table 4:** Statistical analysis of the stranded and necropsied cetaceans 2000–2017 (*n* = 540), focus on traumatic intra-interspecific interaction between cetaceans (*n* = 24).

	**Overall *N* = 540**	**Other cause of death *N* = 408**	**Social traumatic interaction *N* = 24**	***P*-value**
***Species***				0.111
*Balaenoptera acutorostrata*	6 (1.1)	5 (1.2)	0 (0.0)	
*Balaenoptera borealis*	3 (0.6)	2 (0.5)	0 (0.0)	
*Balaenoptera edeni*	2 (0.4)	2 (0.5)	0 (0.0)	
*Balaenoptera physalus*	6 (1.1)	4 (1.0)	0 (0.0)	
*Delphinus delphis*	55 (10.2)	47 (11.5)	3 (12.5)	
*Globicephala macrorhynchus*	45 (8.3)	28 (6.9)	8 (33.3)	
*Grampus griseus*	13 (2.4)	10 (2.5)	0 (0.0)	
*Kogia breviceps*	29 (5.4)	20 (4.9)	3 (12.5)	
*Kogia sima*	7 (1.3)	5 (1.2)	0 (0.0)	
*Lagenodelphis hosei*	4 (0.7)	3 (0.7)	0 (0.0)	
*Megaptera novaeangliae*	2 (0.4)	2 (0.5)	0 (0.0)	
*Mesoplodon bidens*	2 (0.4)	1 (0.2)	0 (0.0)	
*Mesoplodon densirostris*	8 (1.5)	7 (1.7)	0 (0.0)	
*Mesoplodon europaeus*	11 (2.0)	4 (1.0)	2 (8.3)	
*Mesoplodon mirus*	1 (0.2)	0 (0.0)	0 (0.0)	
*Orcinus orca*	1 (0.2)	1 (0.2)	0 (0.0)	
*Phocoena phocoena*	1 (0.2)	1 (0.2)	0 (0.0)	
*Physeter macrocephalus*	32 (5.9)	21 (5.1)	0 (0.0)	
*Pseudorca crassidens*	2 (0.4)	2 (0.5)	0 (0.0)	
*Stenella coeruleoalba*	105 (19.4)	81 (19.9)	3 (12.5)	
*Stenella frontalis*	102 (18.9)	83 (20.3)	3 (12.5)	
*Stenella longirostris*	3 (0.6)	3 (0.7)	0 (0.0)	
*Steno bredanensis*	22 (4.1)	20 (4.9)	0 (0.0)	
*Tursiops truncatus*	42 (7.8)	33 (8.1)	1 (4.2)	
*Ziphius cavirostris*	36 (6.7)	23 (5.6)	1 (4.2)	
***Diving behavior***				0.003
Shallow diver	356 (65.9)	289 (70.8)	10 (41.7)	
Deep diver	184 (34.1)	119 (29.2)	14 (58.3)	
***Coast***				0.014
El Hierro y La Palma	14 (2.6)	8 (2.0)	0 (0.0)	
La Gomera y Tenerife	172 (31.9)	131 (32.1)	16 (66.7)	
Gran Canaria	149 (27.6)	113 (27.7)	3 (12.5)	
Fuerteventura y Lanzarote	205 (38.0)	156 (38.2)	5 (20.8)	
***Age***				0.094
Neonate/calf	142 (26.3)	107 (26.2)	11 (45.8)	
Juvenil/subadult	161 (29.9)	121 (29.7)	4 (16.7)	
Adult	236 (43.8)	180 (44.1)	9 (37.5)	
***Body condition***				0.044
Poor/very poor	166 (34.7)	145 (37.2)	3 (15.0)	
Good/fair	312 (65.3)	245 (62.8)	17 (85.0)	
***Sex***				0.731
Female	254 (48.2)	187 (46.4)	12 (50.0)	
Male	273 (51.8)	216 (53.6)	12 (50.0)	
***Mature categories***				0.768
Immature	261 (49.2)	199 (49.0)	12 (52.2)	
Mature	269 (50.8)	207 (51.0)	11 (47.8)	

*Categorical variables are expressed as frequencies and percentages, in brackets, and were compared, as appropriate, using the Chi-square (χ2) test or the exact Fisher test. Statistical significance was set at p < 0.05*.

Regarding the most affected species, the short-finned pilot whale, 45 individuals stranded in 18 years, and 17.8% died due to intra-interspecific interaction (8/45). It was remarkable the high prevalence of social traumatic interaction in two infrequent stranded species: Gervais' beaked whale and pygmy sperm whale, with 18.2% (2/11) and 10.3% (3/29) of the necropsied individuals affected, respectively.

#### Diving Behavior

Although fewer deep-diving cetaceans were necropsied [34.1%; 184/540] compared to shallow diving cetaceans [65.9%; 356/540], more deep divers presented with traumatic intra-interspecific interaction [58.3%; 14/24] (Table 1). Comparing the affected animals with the number of necropsies of each group, 7.6% of deep divers (14/184) presented this pathological entity, while only 2.8% of shallow divers (10/356) were affected. This difference was found statistically significant (*p* = 0.003) (Table 4).

#### Coast

**Figure 3:**
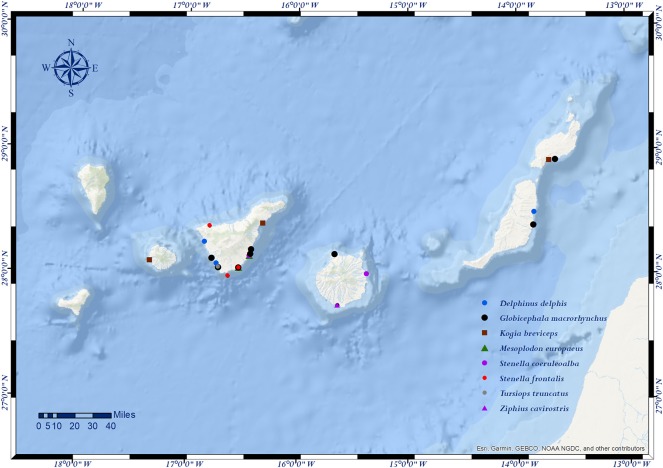
Locations (dots) of cetaceans stranded in the Canary Islands with evidence of social traumatic intra-interspecific interaction (*n* = 24).

The prevalence of strandings per island during the study period was of 38% in Fuerteventura-Lanzarote (205/540), 31.9% in La Gomera-Tenerife (172/540), 27.6% in Gran Canaria (149/540), and 2.6% in El Hierro-La Palma (14/540). However, we found more animals affected by traumatic intra-interspecific interaction in La Gomera-Tenerife [66.7%; 16/24] (Table 1). None of the animals stranded in the western islands of El Hierro-La Palma were affected by this entity. The prevalence of traumatic intra-interspecific interaction between the different coasts was statistically significantly different (*p* = 0.014) (Table 4 and Figure 3).

#### Age

The percentage of necropsied animals for each age class were of 43.7% adults (236/540), 29.8% juveniles/subadults (161/540), and 26.3% neonates/calves (142/540). The age class of one animal could not be determined. In contrast, the age class most affected by traumatic intra-interspecific interactions were neonates/calves with 7.7% (11/142), followed by adults with 3.8% (9/236), and juveniles/subadults with 2.5% (4/161) (Table 1). No statistically significant differences (*p* = 0.094) were found between age classes (Table 4).

#### Body Condition

The prevalence of cetacean's body condition during the study period were 65.3% good/fair (312/540) and 34.7% poor/very poor (166/540). In 62 cetaceans the body condition could not be determined due to advanced decomposition state. This difference was higher in this entity, in which 70.8% (17/24) of affected cetaceans were in good/fair body condition and only 12.5% presented poor/very poor body condition. This difference was found statistically significant (*p* = 0.044) (Table 4).

#### Other Variables With No Statistical Significance (Sex, Mature, and Temporality)

In our study, no statistically significant differences were found in the prevalence of intra-interspecific interaction entity between animals of different sex (*p* = 0.731) nor sexually mature or immature animals (*p* = 0.768) (Table 4). Finally, regarding the temporality of stranding events, no trend was detected. The yearly average occurrence of intra-interspecific interactions was 1–2 animals per year (24 cases over 18 years).

### Traumatic Death Due to an Accident During Predation: Gross and Histological Findings

**Figure 4:**
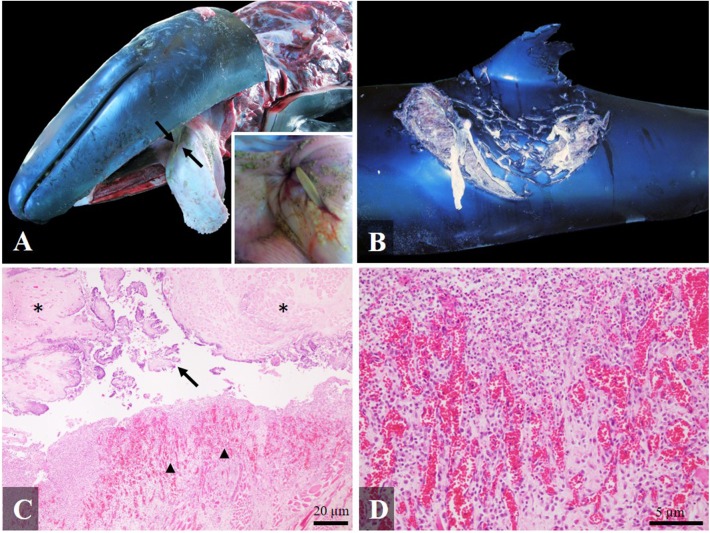
Gross and histological findings (hematoxylin-eosin) in a false killer whale (case 14) with fatal interaction with a stingray: **(A)** Full-thickness perforating glossitis with an intralesional spine of stingray visible on the dorsal surface of the tongue (arrows). Inset: Detail of the spine's tip; **(B)** Severe antemortem shark bites on the dorsal fin and dorsum, affecting skeletal muscle; **(C)** Necrotizing and pyogranulomatous glossitis, with acute degenerative changes in the skeletal muscle (asterisk), presence of cellular debris in the spine trajectory (arrow) and granulation tissue (arrowhead) ×4; **(D)** Detail of the granulation tissue ×20.

In three cases interspecific interaction with potential prey resulted in fatalities. A juvenile false killer whale (case 14) in very poor body condition presented a fatal interaction with a stingray. The main necropsy finding was a full-thickness perforating traumatic necrotizing and granulomatous glossitis and stomatitis involving soft palate (Figure 4A), previously briefly reported by Díaz-Delgado et al. (4). In addition, shark bites along the dorsal fin on both sides of the body with a mild inflammatory reaction (the edges of the wound were enlarged and retracted and the exposed tissue was covered by scarce granulation tissue and fibrin), indicating an antemortem interaction was observed (Figure 4B). Histologically, the perforation of the tongue presented cellular debris, bacteria, neutrophils and necrotic changes in skeletal muscle (hypercontraction and hyalinization of myofibers), surrounded by scarce granulation tissue with associated angiogenesis and fibrosis (Figures 4C,D). Severe changes were observed also in the *longissimus dorsi*, with multifocal polyphasic myocyte degeneration and necrosis: segmental myonecrosis associated with the stress stranding syndrome, atrophy due to emaciation, and myositis due to the shark bites (7).

The other cases were two Risso's dolphins (cases 17 and 20) with decompression sickness (44). The first animal (case 17) was an immature male with good body condition that presented multifocal lacerations consistent with a live stranding as well as social intra-interspecific interaction marks. The distal segment of a squid tentacle was observed partially fixed on the mandible skin and ring-associated marks were described on the oral cavity and cervical skin. Two additional 110 cm-long tentacles ran through the esophagus associated with acute hemorrhages on the cranial part. A partially digested large squid *Ommastrephes bartramii* (LeSueur, 1821) was found on the keratinized gastric compartment with abundant dark liquid. Also, non-collapsed lungs with rib marks, marked pleural lymphangiectasia and pulmonary edema filling the trachea were present.

The second animal (case 20) was a mature female with external signs of live stranding in poor body condition, but presented a large undigested squid in the keratinized gastric compartment. Additionally, the left lung was partially collapsed and a 5 cm lung rupture with associated fibrosis and hemorrhage were described on the dorsal aspect. Gross and histologically, both animals presented generalized round to oval intravascular spaces consistent with gas bubbles. Gas analysis of both animals confirmed a systemic decompression sickness (45, 46).

## Discussion

### Social Traumatic Interactions Between Cetaceans

#### Gross Findings

In this retrospective study, we focused on 24 cases with severe traumatic lesions highly compatible with intra-interspecific interactions (5, 6, 37). We found that blunt traumas were multifocal, with fractures and bruises in different locations affecting both body sides, and in some cases, associated with multiple severe tooth-rake marks. Other findings, such as tracheal edema, undigested food in the stomach, pulmonary perforation, and both, hemothorax and hemoabdomen, were also described.

It is important to emphasize that except for the acute rake marks, none of the lesions described could be considered pathognomonic for this entity, as they can be produced during other traumatic events (i.e., vessel strikes, fishing interactions, or live stranding). Undeniably, severe acute tooth-rake marks are indicative of aggressive interaction with other cetaceans. However, the absence of tooth-rake marks does not rule out this entity. For example, killer whales striking with their snouts produced internal injuries in other cetaceans without causing external wounds (24). Also, blunt-force traumas can be produced by vessel strikes with the hull. However, in vessel strikes, contusions are mostly unidirectional and located on the dorsum (38). Therefore, we should always perform differential diagnoses.

Other traumatic events include fisheries interaction and live strandings. In bycatch cases, the presence of external net marks is a diagnostic key, frequently associated with the presence of undigested food in the stomach or esophagus, red eyes, and disseminated gas bubbles (46). Finally, during active stranding events, multifocal erosions, and lacerations of variable extent can be produced, mainly in ventral parts of the body, flanks, pectoral fins, tail fluke, and rostrum (4). In our study, we discarded cases in which traumatic etiologies other than inter or intraspecific interactions could not be ruled out.

#### Histological Findings

There was a high prevalence of acute monophasic degeneration of the skeletal muscle and myocardium in the cases diagnosed with intra- interspecific interaction. In fewer cases we observed pigmentary tubulonephrosis, intracytoplasmic hepatocellular hyaline globules, bronchiolar sphincter contraction, and corticomedullary adrenal hemorrhages. These findings have been previously reported in association with stressful agonal events or in severely polytraumatized animals. For example: segmental myodegeneration and contraction band necrosis have been described before in traumatized stranded cetaceans (7); pigmentary tubulonephrosis, as well as hyaline casts, have been associated with capture myopathy in live strandings (47); and the presence of vacuoles, known as hyaline globules, in the hepatocytes and myocardiocytes have been previously reported in acute stressful deaths (4, 41, 48, 49). Thus, histological determination of agonal changes (in skeletal and cardiac muscle, kidneys, lungs, liver, and adrenal glands) can support gross evidence in cases suspected of traumatic intra-interspecific interaction.

### Statistical Analysis

#### Species

The Canary waters contain one-third of the cetacean species recorded around the world (Banco de Datos de Biodiversidad de Canarias). In our study, the short-finned pilot whale was the most affected species. The south-west coast of Tenerife holds a resident population of short-finned pilot whales in deep waters from 800 to 2000 m (50). Oremland 2010 supports the hypothesis of intraspecific interaction due to sexual competition in this species. High prevalence of mandibular fractures, 54% (27/50), was described in individuals of both sexes [females with 47% (17/36) and males with 71% (10/14)] of two mass stranding events in North and South Carolina (51). In that research, the prevalence of mandibular fractures increased with the length of the animal, suggesting that the animals may use their heads during fights. In our study, the prevalence of mandibular fractures, as well as cranial fractures, was low [25% (2/8)], while multifocal contusions (6/8) associated with hemothorax (4/8) were more prevalent.

The study of tooth-rake marks allowed us to determine fatal interaction with killer whales in three cetacean species, including a Cuvier's Beaked whale, pygmy sperm whale and short-finned pilot whale. Killer whales have been observed predating *Mesoplodon* spp. (28) and feeding on fresh carcasses of Cuvier's beaked whales which they probably killed (52). Also, dwarf sperm whales (*Kogia sima*) have been seen attacked by killer whales in the Bahamas (53). There is also indirect evidence (remnants in the stomach) of killer whales feeding on short-finned pilot whales and pygmy sperm whales, although this evidence did not allow determination of whether the feeding behavior was predation or carrion (24).

In the Canary Islands, killer whales have been sighted in spring and summer, associated with the presence of tuna. Few aggressive encounters between killer whales and short-finned pilot whales have been observed in the Canaries. In one of them, a huge group of short-finned pilot whales was recorded pursuing and deterring a group of killer whales from their territory at the South of La Gomera (http://www.rtvc.es/noticias/video-graban-a-un-grupo-de-calderones-persiguiendo-a-una-familia-de-orcas-en-la-189459.aspx#.XcBOq5r7TIV). On the other hand, a group of killer whales was seen attacking and feeding on two short-finned pilot whales (https://www.antena3.com/noticias/sociedad/un-grupo-de-orcas-atacan-a-dos-calderones-en-tenerife_201807305b5eca210cf267fe6b5e3054.html) in the South of Tenerife. Killer whales have also been observed feeding on two fresh calf carcasses of beaked whales (https://www.elmundo.es/elmundo/2013/08/02/natura/1375440241.html), presumably *Mesoplodon* spp, and also on a live pygmy sperm whale (https://www.youtube.com/watch?v=8Dxkg0n4rRE) in the Canary Islands.

Tooth-rake marks compatible with bottlenose dolphins were present in two species: short-beaked common dolphin and Atlantic spotted dolphin. Bottlenose dolphins are residents in the Canary Islands (31), and are well-known for interacting aggressively worldwide within these species with different motivations (37). Either way, bottlenose dolphins are well-known for their aggressive interaction with other species (37, 54, 55). Additionally, two females of stripped dolphin presented intraspecific tooth-rake marks. One of them (case no 4) presented clear fresh tooth-rake marks surrounding the genital area, which is highly related with sexual aggressive behavior (1).

Summarizing, deep divers were more attacked by killer whales while shallower species were mainly attacked by bottlenose dolphins in our study.

#### Diving Behavior

Our results show that deep divers are more prone to intra-interspecific interactions than shallow divers unlike consulted references, in which more encounters have been published about shallow-diving species (e.g., 24, 37, 26, 27, 28). On the other hand, the Canary Islands are known for the presence of deep-diving species, at least one-third of the species recorded. Some deep divers require time resting on the surface, predisposing them to vessel strikes (56), but also potentially making them vulnerable to attacks from predatory cetaceans like killer whales.

#### Coast

The prevalence of intra-interspecific cases was highest on Tenerife and La Gomera coast. Open water observations in the archipelago support that resident populations of bottlenose dolphins and short-finned pilot whales coexist with the Atlantic spotted dolphin, the short-beaked common dolphin, and the rough-toothed dolphins in La Gomera waters (31). Prey competition due to diet overlap has been postulated to explain cases of lethal interactions between bottlenose dolphins and harbor porpoises (*Phocoena phocoena*) (21). Behavioral observational studies suggest that traumatic interactions between these species were rare in this area, as cetaceans occupying the same living space are separated by their prey specialization (31). However, as evidenced by our results, fatal traumatic interactions do occur, involving especially bottlenose dolphins and killer whales when present.

#### Age Class

In our study, 50% of the affected individuals were neonates or calves, although this group represented only 25% of the total studied cases. Three affected calves were short-beaked common dolphins and presented rake marks compatible with the bottlenose dolphin. This species is well-known for infanticide (1, 2) and attacking smaller sized cetaceans such as porpoises (57). In this way, infanticide in bottlenose dolphins, in which neonates (1–1.3 m, 12–25 kg) have similar sizes to adult harbor porpoises (0.74–1.66 m) (57), may be explained as fight practice (6).

Additionally, we found three pregnant pygmy sperm whales with intra-interspecific interactions. Interestingly, 85.7% (6/7) of mature female pygmy sperm whales stranded in the Canary Islands were pregnant. The gestation period in this species is about 9.5–11 months and the length at birth around 1 m (58, 59). In Pinedo (60), some cases of stranded females with calves and fetuses were collected. Thus, concurrent lactation and pregnancy in this species is not unusual (58, 59) and therefore a higher percentage of the female population might be pregnant at one time compared to other species.

#### Body Condition

This variable was statistically significant in this study as most of the cases presented good/fair body condition. However, the ecological and pathological meaning of this result remains unknown as good/fair body condition is a common finding (65%) in cetaceans in this archipelago, based on our stranding data.

### Traumatic Death Due to an Accident During Predation

Predation also has its risks: dislocation of the larynx has been reported in bottlenose dolphins due to the ingestion of a black margate (*Anisotremus surinamensis*) (61) and a beheaded sheepshead (*Archosargus probatocephalus*) (62), asphyxia due to obstruction of the airway was reported in long-finned pilot whales (*Globicephala melas*) with a common sole (*Solea solea*) (63) and a European eel (*Anguilla anguilla*) (64), and inflammation of the throat produced by ingested fish species with strong dorsal spines lead to the death of some bottlenose dolphins (65, 66).

In three of our cases, the death of the animals was directly associated with an accident during predation. A false killer whale (case 14) presented an intralesional stingray spine in the tongue causing severe chronic perforating glossitis and stomatitis (4). This animal also presented with severe muscular atrophy due to starvation, muscular degenerative changes due to an active stranding, and antemortem shark bites (7). Fatal interactions between dolphins and stingrays have been well-documented. In bottlenose dolphins, abdominal and lateral chest perforations, organ punctures (in liver, pancreas, esophagus, stomach, heart, lung, and trachea) (66, 67), caudal vena cava perforations (68), and an intestinal concretion with intralesional stingray spines (69) have all been reported. In addition, an accidental finding of a stingray spine was reported in the right scapula of a bottlenose dolphin from South Carolina, USA (70). Also, there is a report of esophageal perforation by a stingray spine in a killer whale (71). To our knowledge this was the first report of a false killer whale fatal predation accident with a stingray.

Finally, two Risso's dolphins (cases 17 and 20) presented fatal interaction with large squids and died from decompression sickness (44). The dolphins presented evidence or struggling/fighting with the squid. In consulted references, an adult shallow Indo-Pacific bottlenose dolphin (*Tursiops aduncus*) died by suffocation due to fatal octopus ingestion in Bunbury, Western Australia (72). As in case 17, some octopus' arms protruded from the mouth of the dolphin and suckers were firmly adhered to the caudal tongue, pharynx, and the esophageal mucosa, producing red-purple circular umbilicated lesions (72). Also, hyperinflated lungs with marked rib impressions were described. In shallow depths of the same area, adult Indo-Pacific bottlenose dolphins have been seen handling octopus (73). Poorly handled prey items can be fatal (73). In Stephens et al. (72), the “goosebeak” larynx of the dolphin was displaced, compressed ventrally, and obstructed with a remaining tentacle. In our cases 17 and 20, no larynx dislocation was present, but struggling with the squid may have resulted in severe alterations in the diving profile and physiologically induced formation of gas emboli (44).

## Conclusions

This is the first study with a focus on traumatic intra-interspecific interaction between cetaceans in the Canary Islands. The full anatomopathological study is necessary to reach a traumatic intra-interspecific interaction diagnosis and to differentiate it from other traumatic etiologies. We described acute severe tooth-rake marks compatible with killer whales and bottlenose dolphins in five species (pigmy sperm whale, Cuvier's beaked whale, short-finned pilot whale, short-beaked common dolphin, and Atlantic spotted dolphin), and intraspecific aggressive tooth-rake marks in stripped dolphin. The aggressor species was identified based on inter-tooth distances. Deep-divers, in good body condition, and/or stranded nearby La Gomera and Tenerife were more affected by social traumatic interaction in this study.

We encourage open water observations and further pathological studies to better understand the origin of this natural behavior, sometimes lethal.

## Data Availability Statement

All datasets generated for this study are included in the article/supplementary files.

## Ethics Statement

Required permission for the management of stranded cetaceans was issued by the environmental department of the Canary Islands' Government and the Spanish Ministry of Environment. Experiments on live animals were not performed.

## Author Contributions

RP-L, YB, and MAr: conceptualization. AF, MAr, MAn, ES, YB, MT, JD, AX, JD-D, and RP-L: sampling and diagnosis of the cause of death of each animal. PS, RP-L, and YB: data analyses. JD: map editing. RP-L and MR: image editing. RP-L: writing. YB, MAr, and MR: supervision. All authors: review and editing.

### Conflict of Interest

The authors declare that the research was conducted in the absence of any commercial or financial relationships that could be construed as a potential conflict of interest.
